# FOXM1 confers to epithelial-mesenchymal transition, stemness and chemoresistance in epithelial ovarian carcinoma cells

**DOI:** 10.18632/oncotarget.2957

**Published:** 2014-12-10

**Authors:** Wen-Tai Chiu, Yu-Fang Huang, Huei-Yu Tsai, Chien-Chin Chen, Chang-Hwa Chang, Soon-Cen Huang, Keng-Fu Hsu, Cheng-Yang Chou

**Affiliations:** ^1^ Department of Biomedical Engineering, National Cheng Kung University, Tainan, Taiwan; ^2^ Institute of Basic Medical Sciences, National Cheng Kung University, Tainan, Taiwan; ^3^ Department of Obstetrics and Gynecology, National Cheng Kung University Hospital, College of Medicine, National Cheng Kung University, Tainan, Taiwan; ^4^ Cancer Research Center, National Cheng Kung University Hospital, College of Medicine, National Cheng Kung University, Tainan, Taiwan; ^5^ Department of Pathology, Chia-Yi Christian Hospital, Chiayi, Taiwan; ^6^ Department of Cosmetic Science, Chia Nan University of Pharmacy and Science, Tainan, Taiwan; ^7^ Department of Obstetrics and Gynecology, Chi Mei Medical Center, Liouying Campus, Tainan, Taiwan

**Keywords:** ovarian cancer, FOXM1, β-CATENIN, chemoresistance, stemness

## Abstract

Chemoresistance to anti-cancer drugs substantially reduces survival in epithelial ovarian cancer. In this study, we showed that chemoresistance to cisplatin and paclitaxel induced the epithelial-mesenchymal transition (EMT) and a stem cell phenotype in ovarian cancer cells. Chemoresistance was associated with the downregulation of epithelial markers and the upregulation of mesenchymal markers, EMT-related transcription factors, and cancer stem cell markers, which enhanced invasion and sphere formation ability. Overexpression of FOXM1 increased cisplatin-resistance and sphere formation in cisplatin-sensitive and low FOXM1-expressing ovarian cancer cells. Conversely, depletion of FOXM1 via RNA interference reduced cisplatin resistance and sphere formation in cisplatin-resistant and high FOXM1-expressing cells. Overexpression of FOXM1 also increased the expression, nuclear accumulation, and activity of β-CATENIN in chemoresistant cells, whereas downregulation of FOXM1 suppressed these events. The combination of cisplatin and the FOXM1 inhibitor thiostrepton inhibited the expression of stem cell markers in chemoresistant cells and subcutaneous ovarian tumor growth in mouse xenografts. In an analysis of 106 ovarian cancer patients, high FOXM1 levels in tumors were associated with cancer progression and short progression-free intervals. Collectively, our findings highlight the importance of FOXM1 in chemoresistance and suggest that FOXM1 inhibitors may be useful for treatment of ovarian cancer.

## INTRODUCTION

### Chemoresistance is a major obstacle in ovarian cancer therapy

Ovarian cancer is the second most common gynecological malignancy and a major contributor to death from cancer in women [1]. While the initial response to first-line therapy (cytoreductive surgery and combined platinum-paclitaxel chemotherapy) is usually effective, most cancers recur, are chemotherapy-resistant, and result in the death of the patient. The mechanism responsible for chemoresistance in epithelial ovarian cancer (EOC) remains little understood, and overcoming chemoresistance is an important goal in cancer therapy [2].

### Chemoresistance of cancer stem cells is critical for cancer therapy

Accumulated evidence has led to the hypothesis that solid tumors contain a small subpopulation of cancer stem cells (CSCs), which are self-renewing and responsible for tumor maintenance, metastasis, and possibly resistance to chemotherapy and radiotherapy [3, 4]. CSCs are enriched in tumors and cultured cancer cells treated with chemotherapeutic drugs and, therefore, are resistant to chemotherapy. In clinical practice, optimal chemotherapy kills most of the cells in solid tumors. However, CSCs acquire changes that confer drug resistance and hence a selection advantage, eventually generating a new population of chemoresistant cancer cells [5, 6]. Such changes include cell quiescence and expression of membrane transporters that pump drugs out of cells [7]. As a result, CSCs survive chemotherapy and regenerate the tumor. Targeting treatments to CSCs would thus be a logical way of overcoming chemoresistance and improving the outcome of cancer treatment. However, the mechanisms involved in CSC chemoresistance are complex and not clearly defined.

### FOXM1 regulates stemness and chemoresistance in cancers

FOXM1, a member of the forkhead transcription factor family [8], is required for cell cycle progression [9-11], apoptosis [12], angiogenesis [13], and DNA damage repair [14]. In addition, aberrant expression of *FOXM1* is linked to tumorigenesis and chemoresistance. A systemic analysis of gene expression profiles in microarrays showed that *FOXM1* mRNA was overexpressed in nearly every tumor analyzed, including ovarian tumors [15]. Other studies showed that FOXM1 and its downstream DNA damage repair targets BRCA1, BRCA2, and XRCC1 increased cisplatin resistance in different types of cancer cells [16-18], as well as herceptin and paclitaxel [19] resistance in breast cancer cells. FOXM1 is highly expressed in multipotent progenitor cells and inhibits their differentiation [20, 21] and, as more recently reported, upregulates the expression of the pluripotent genes *OCT-4*, *NANOG*, and *SOX-2* when overexpressed [22]. FOXM1 also participates in an early oncogenic pathway that predisposes cells to tumorigenesis by expanding the stem/progenitor cell compartment [23]. These findings suggest a critical involvement of FOXM1 in the maintenance of stem cell pluripotency.

### FOXM1 regulates β-CATENIN-mediated stemness and tumorigenesis

The WNT network influences a wide range of biological processes including developmental cell fate, cell polarity and adhesion, tumorigenesis, and apoptosis. Numerous studies suggest that it promotes tumorigenesis by maintaining stem and CSC populations [24, 25]. The key feature of WNT signaling activation is β-CATENIN nuclear localization. Reciprocal regulation of the WNT/β-CATENIN pathway and FOXM1 has been reported recently. Mirza *et al.* showed that FOXM1 directly binds the human *β-CATENIN* promoter and upregulates its expression in endothelial cells [26]. On the other hand, Zhang *et al.* found that WNT3A increases the abundance of nuclear FOXM1, which interacts with and promotes the nuclear accumulation and transcriptional activity of β-CATENIN in tumor cells [27]. Moreover, both proteins formed a complex with the TCF transcription factors on the promoters of WNT/β-CATENIN target genes. These findings show that FOXM1 controls the expression of WNT target genes by interacting with β-CATENIN or its promoter.

### FOXM1 inhibitors are effective against tumors

FOXM1 is an attractive molecular target for anticancer therapies because it interacts with numerous signaling pathways and it is expressed by many solid tumors. FOXM1 inhibitors such as the thiazole antibiotics siomycin A and thiostrepton [28, 29], induce the apoptosis of many types of cancer cells and have been approved by the Federal Drug Administration for animal use. Treatment of human cancer cell lines with siomycin A or thiostrepton not only inhibits FOXM1 activity but also its expression [30]. Importantly, FOXM1 inhibitors have no effect on FOXM1 expression in or the proliferation of nontransformed cells and exert minimal toxicity against noncancer cells.

In the present study, we show that FOXM1 is a critical regulator of the epithelial-mesenchymal transition (EMT), stemness, and chemoresistance in ovarian cancer cells. WNT/β-CATENIN signaling required FOXMI, as did the growth of ovarian cancers. A clinical investigation established a relationship between FOXM1 expression and unfavorable outcomes in EOC patients, thus validating our *in vitro* findings.

## RESULTS

### Establishment of chemoresistant sublines of ovarian cancer IGROV1 cells

**Figure 1:**
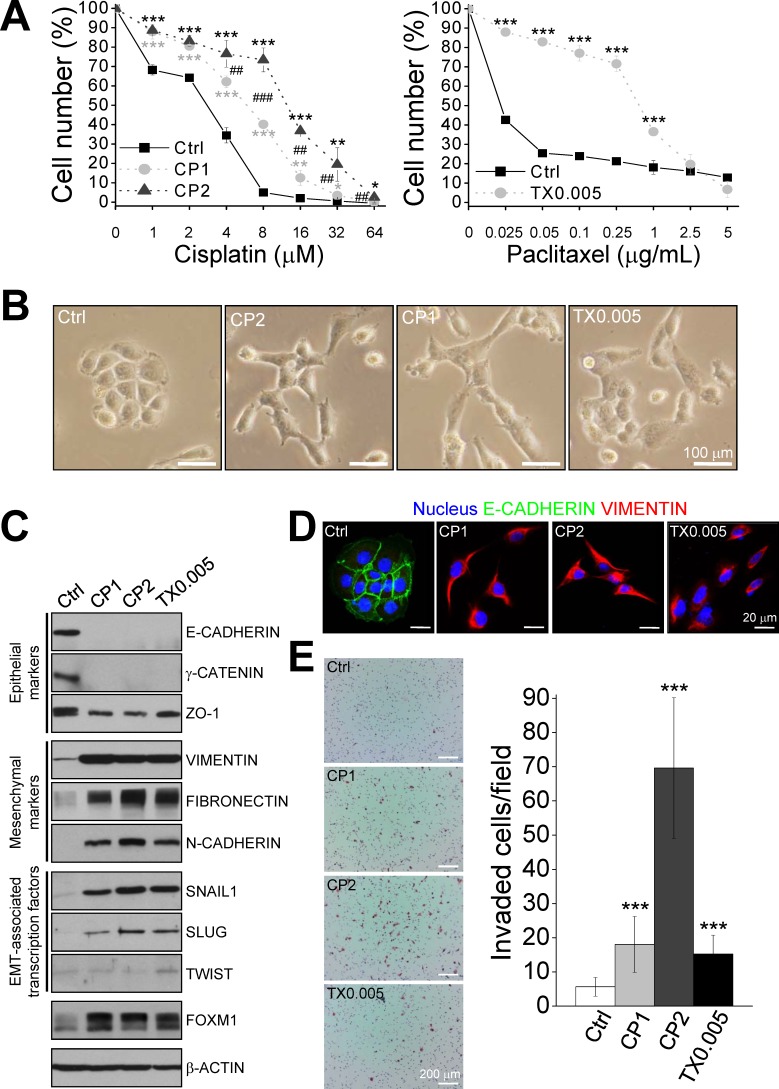
Chemoresistant IGROV1 sublines exhibit characteristics of the epithelial-mesenchymal transition (EMT) IGROV1 cells resistant to 1 μM cisplatin (CP1), 2 μM cisplatin (CP2), and 0.005 μg/mL paclitaxel (TX0.005) were isolated. (A) The IC_50_ values of the parental (Ctrl) and resistant cell lines were determined using MTT assays. (B) Phase contrast images of parental and chemoresistant cells. Scale bars, 100 μm. (C) Amounts of the indicated proteins were determined via western blotting. (D) Immunofluorescence staining of nuclei, E-CADHERIN, and VIMENTIN. Images were taken using a confocal microscope under excitation at 405 nm, 488 nm, or 543 nm. Scale bars, 20 μm. (E) *In vitro* transwell invasion assay. Left panel, representative photomicrographs of cells that penetrated a Matrigel-coated filter. Scale bars: 200 μm. Right panel, invasive cells were counted in 15 random fields on the lower surface of the filters and are expressed as invaded cells per field. Each bar represents mean ± standard error of the mean from two independent experiments. *: significant difference between chemoresistant and parental cells (A, E); #: significant difference between CP1 and CP2 cells (A). *: *P* < 0.05; **^, ##^: *P* < 0.01; ***^, ###^: *P* < 0.001 (A, E).

To elucidate the underlying mechanisms of chemoresistance in ovarian cancer, human ovarian cancer sublines resistant to cisplatin or paclitaxel were established. As shown in Fig. 1A, the IGROV1 sublines CP1 and CP2 were more resistant to cisplatin than parental cells (IC_50_ values were 5.88, 12.57, and 2.78 μM, respectively; *P* = 0.002, Kruskal-Wallis test). Similarly, the IGROV1 subline TX0.005 was more resistant to paclitaxel than parental cells (IC_50_ values were 0.60 μg/mL and 0.02 μg/mL, respectively; *P* = 0.002). Compared with parental cells, the drug resistant cells had an elongated mesenchymal-like morphology and fewer cell-cell junctions (Fig. 1B).

### Chemoresistant ovarian cancer cells have an EMT phenotype and high invasion ability

Mesenchymal markers (VIMENTIN, FIBRONECTIN, and N-CADHERIN) and EMT-associated transcription factors (SNAIL1, SLUG, and TWIST) were upregulated in chemoresistant cells compared with parental cells, whereas epithelial markers (E-CADHERIN, γ-CATENIN, and ZO-1) were down-regulated (Fig. 1C). In addition, FOXM1 expression was markedly increased in chemoresistant cells. Immunofluorescence staining (Fig. 1D) confirmed the changes in VIMENTIN and E-CADHERIN expression observed in western blots and showed that VIMENTIN was expressed in the cytoplasm and E-CADHERIN primarily in cell-cell junctions. Chemoresistant IGROV1 cells exhibited abundant VIMENTIN staining in the cytoplasm and scarce expression of E-CADHERIN. *In vitro* transwell invasion assays showed that chemoresistant IGROV1 cells were more invasive than parental cells (Fig. 1E). Collectively, these data indicate that the chemoresistant cells acquired an EMT phenotype and invasive ability.

### Chemoresistance contributes to the stemness of ovarian cancer cells

**Figure 2:**
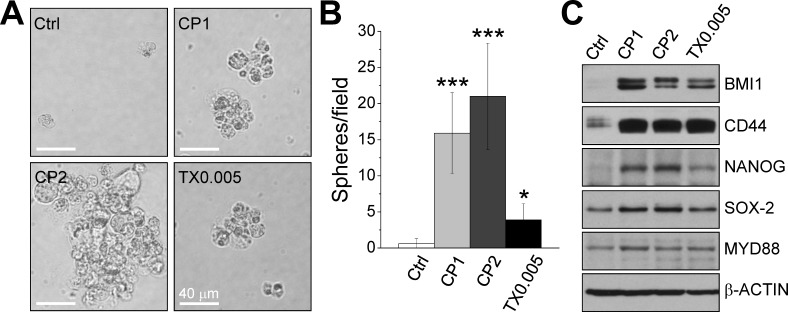
Chemoresistance contributes to stemness The stemness of control (Ctrl), CP1, CP2, and TX0.005 IGROV1 cells was examined via the sphere formation assay and expression of stem cell markers. (A) Representative phase contrast images of suspension spheres. Scale bars, 40 μm. (B) Number of spheres formed in each cell line per low magnification field. Each bar represents mean ± standard error of the mean from three independent experiments and at least 30 different fields. *: significant difference between chemoresistant and parental cells. *: *P* < 0.05; ***: *P* < 0.001. (C) Western blots of stem cell markers and the internal control β-ACTIN.

**Figure 3:**
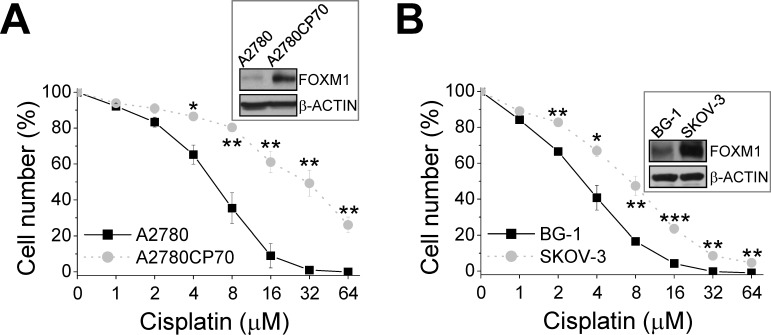
Correlation between FOXM1 levels and cisplatin resistance in ovarian cancer cells (A and B) MTT assays were performed to determine the IC_50_ values for cisplatin in BG1, SKOV-3 (A), A2780, and A2780CP70 (B) cells. The insets show the western blots for FOXM1 and the internal control β-ACTIN. *: significant difference between different cell lines. *: *P* < 0.05; **: *P* < 0.01; ***: *P* < 0.001.

**Figure 4:**
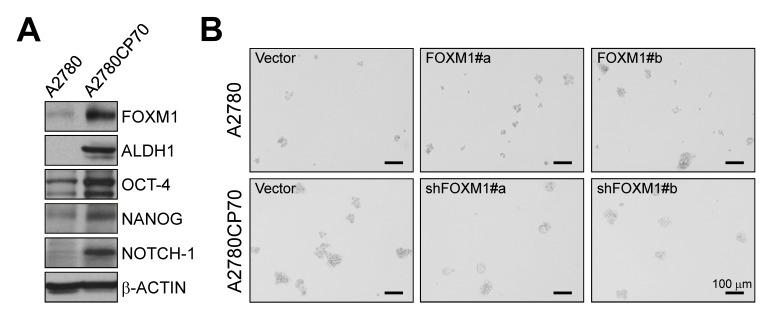
Effects of FOXM1 on stemness in the paired ovarian cancer cell lines A2780 and A2780CP70 (A) Western blots of stem cell markers and the internal control β-ACTIN. (B) Upper panels: A2780 cells were stably transfected with vector alone or vector encoding FOXM1 (FOXM1 #a and #b). Lower panels: A2780CP70 cells were stably transfected with vector alone or vector encoding shFOXM1 (shFOXM1 #a and #b). Representative phase contrast images of the suspension spheres were taken on a widefield microscope. Scale bars, 100 μm.

We examined sphere formation and the expression of stem cell markers in cisplatin-resistant and paclitaxel-resistant IGROV1 cells. Figure 2A shows that chemoresistant cells formed non-adherent spheres, whereas parental cells did not. Cisplatin-resistant cells formed more spheres than paclitaxel-resistant cells (Fig. 2B), and cells selected with a higher dose of cisplatin (CP2) formed larger spheres (> 50 μm) than cells selected with a lower dose of cisplatin (CP1) (Fig. S1). Chemoresistant cells expressed higher amounts of the stem cell markers BMI1, CD44, NANOG, SOX-2, and MYD88 than parental cells (Fig. 2C). We also examined the expression of stem cell markers in the canonical cisplatin-sensitive and cisplatin-resistant ovarian cancer cell limes A2780 (IC_50_ = 5.70 μM) and A2780CP70 (IC_50_ = 30.66 μM) (Fig. 3A). Our results showed that A2780CP70 cells overexpressed several stem cells markers as well as FOXM1 (Fig. 4A).

### FOXM1 enhances cisplatin resistance and sphere formation in ovarian cancer cells

**Figure 5:**
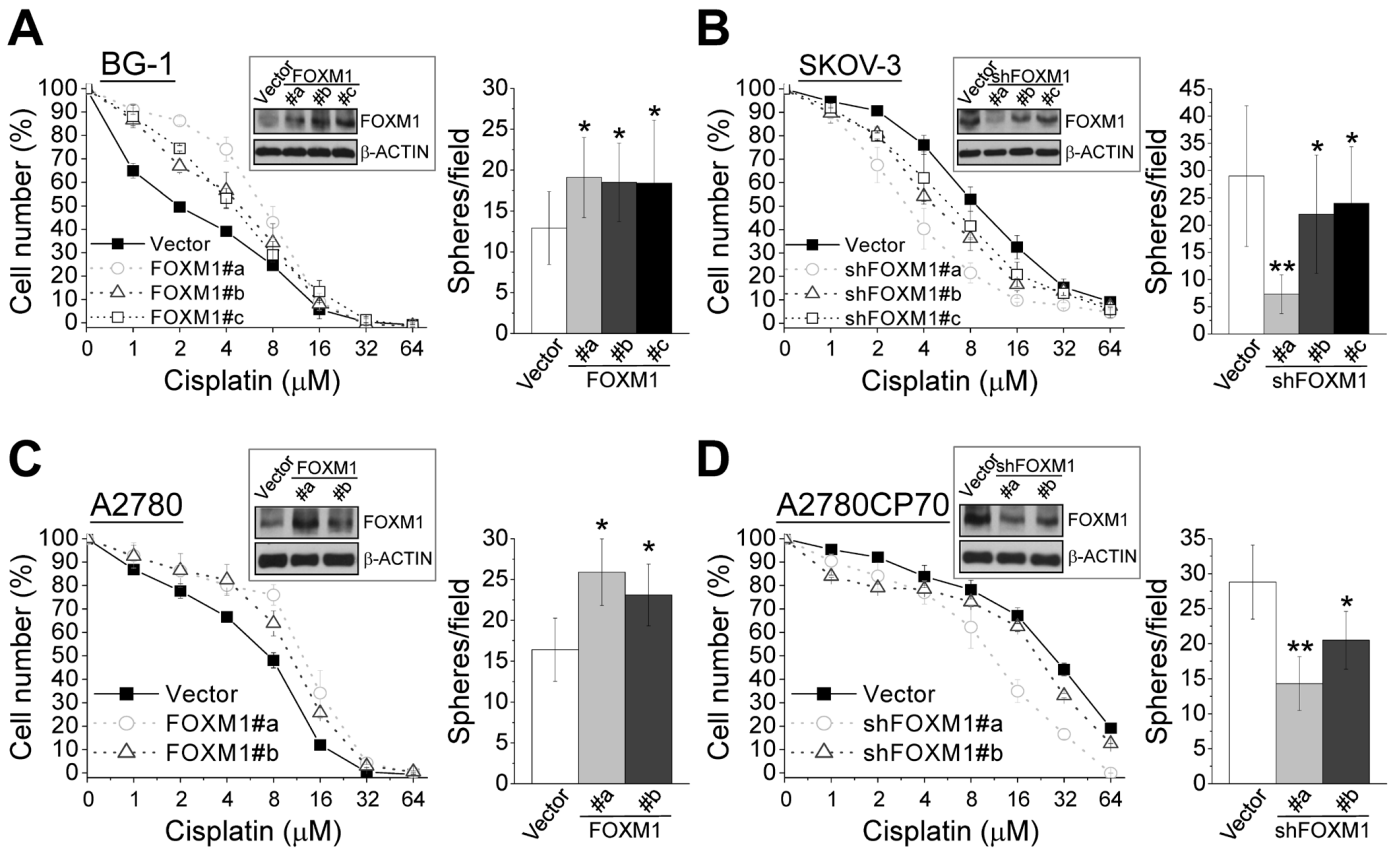
FOXM1 enhances cisplatin resistance and sphere formation in ovarian cancer cells BG-1 (A) and A2780 (C) cells were stably transfected with vector alone or encoding FOXM1 (FOXM1 #a, #b and #c). SKOV-3 (B) and A2780CP70 (D) cells were stably transfected with vector alone or encoding shFOXM1 (shFOXM1 #a, #b, and #c). (A–D) MTT and sphere formation assays were performed. The insets show the western blots for FOXM1 and the internal control β-ACTIN. *: significant difference between FOXM1 overexpressed or silenced cells and vector control cells. *: *P* < 0.05; **: *P* < 0.01.

**Figure 6:**
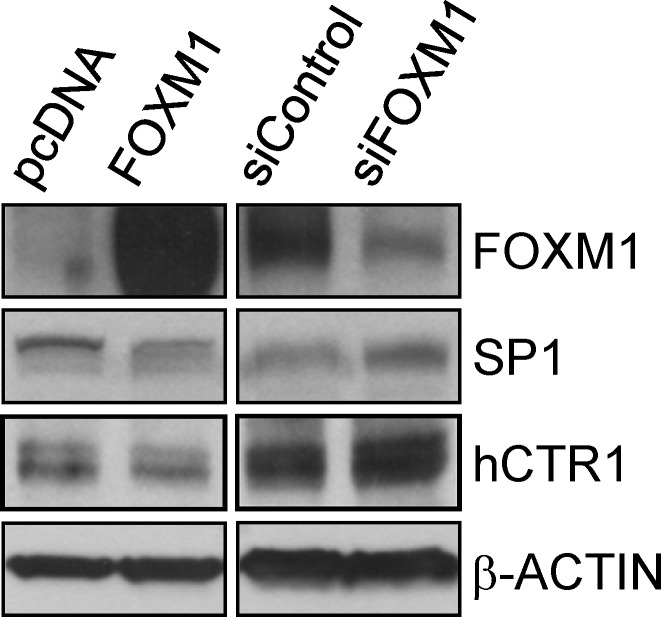
FOXM1 downregulates the expression of SP1 and hCTR1 Western blots of FOXM1, SP1, hCTR1, and the internal control β-ACTIN in Ad293 cells expressing vector alone (pcDNA), vector encoding FOXM1, control siRNA (siControl), or FOXM1 siRNA (siFOXM1).

The higher expression of FOXM1 in chemoresistant cells than parental cells (Figs. 1C and 4A) suggests that FOXM1 may contribute to chemoresistance. To test this premise, we examined FOXM1 expression in two additional ovarian cancer cell lines. BG-1 cells expressed less FOXM1 than SKOV-3 cells (Fig. 3B) and were less resistant to cisplatin (IC_50_ of SKOV-3 = 7.31 μM, IC_50_ of BG-1 = 3.13 μM) (Fig. 3B). We then established three BG-1 stable clones that overexpressed FOXM1 and three stable SKOV-3 clones that expressed FOXM1 shRNA. As shown in Fig. 5A, FOXM1-overexpressing cells (#a, #b, and #c) exhibited higher cisplatin resistance than vector-transfected cells (IC_50_ values were 6.90, 4.83, 4.36, and 1.95 μM, respectively; *P* = 0.016, one-way ANOVA) and higher sphere formation ability. Conversely, SKOV-3 cells depleted of FOXM1 (#a, #b, and #c) were less resistant to cisplatin than vector-transfected cells (IC_50_ values were 3.14, 4.68, 6.01, and 8.82 μM, respectively; *P* < 0.001, one-way ANOVA) and had lower sphere formation ability (Fig. 5B). The effect of FOXM1 on chemoresistance and stemness was also investigated in A2780 and A2780CP70 cells. Overexpression of FOXM1 in A2780 cells (#a and #b) enhanced cisplatin resistance compared with the vector control (IC_50_ values were 12.44, 10.19, and 7.45 μM, respectively; *P* < 0.001, one-way ANOVA) and sphere formation (Figs. 4B and 5C). In contrast, FOXM1 silencing in A2780CP70 cells decreased cisplatin resistance compared with the vector control (IC_50_ values were 10.89, 21.39, and 26.07 μM, respectively; *P* < 0.001, Kruskal-Wallis test) and sphere formation (Figs. 4B and 5D). Of note, the effects of FOXM1 on these responses were dose-dependent. We also determined whether FOXM1 affects the expression of human copper transporter 1 (hCTR1), which transports cisplatin into cells to elicit a cytotoxic effect. Overexpression of FOXM1 in the human embryonic kidney Ad293 cells decreased amounts of hCTR1 and its regulatory transcription factor SP1, while knockdown of FOXM1 via RNA interference increased amounts of hCTR1 and SP1 (Fig. 6). These findings suggest FOXM1 promotes cisplatin resistance by impairing cisplatin uptake.

### FOXM1 promotes β-CATENIN expression, activation, and nuclear localization in chemoresistant ovarian cancer cells

**Figure 7:**
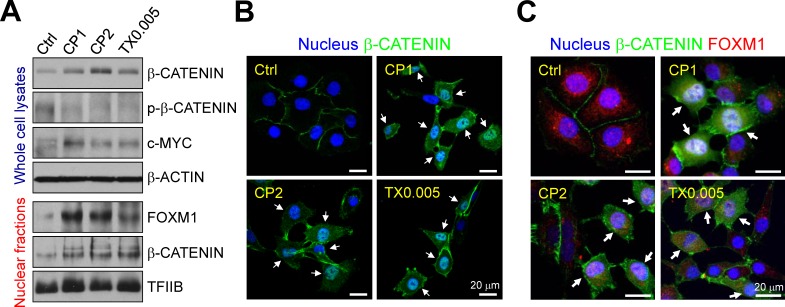
Chemoresistance promotes β-CATENIN expression, activation and nuclear localization (A) Whole cell lysates and nuclear fractions of parental (Ctrl), CP1, CP2, and TX0.005 IGROV1 cells were immunoblotted with antibodies to the indicated proteins; β-ACTIN and TFIIB are internal controls. (B and C) Immunofluorescence staining of the nucleus, β-CATENIN, and FOXM1 in IGROV1 sublines. Representative fluorescent images were taken on a confocal microscope under excitation at 405 nm, 488 nm, or 543 nm. Scale bars, 20 μm. White arrows indicate nuclear β-CATENIN (B) and nuclear co-localization of β-CATENIN and FOXM1 (C).

**Figure 8:**
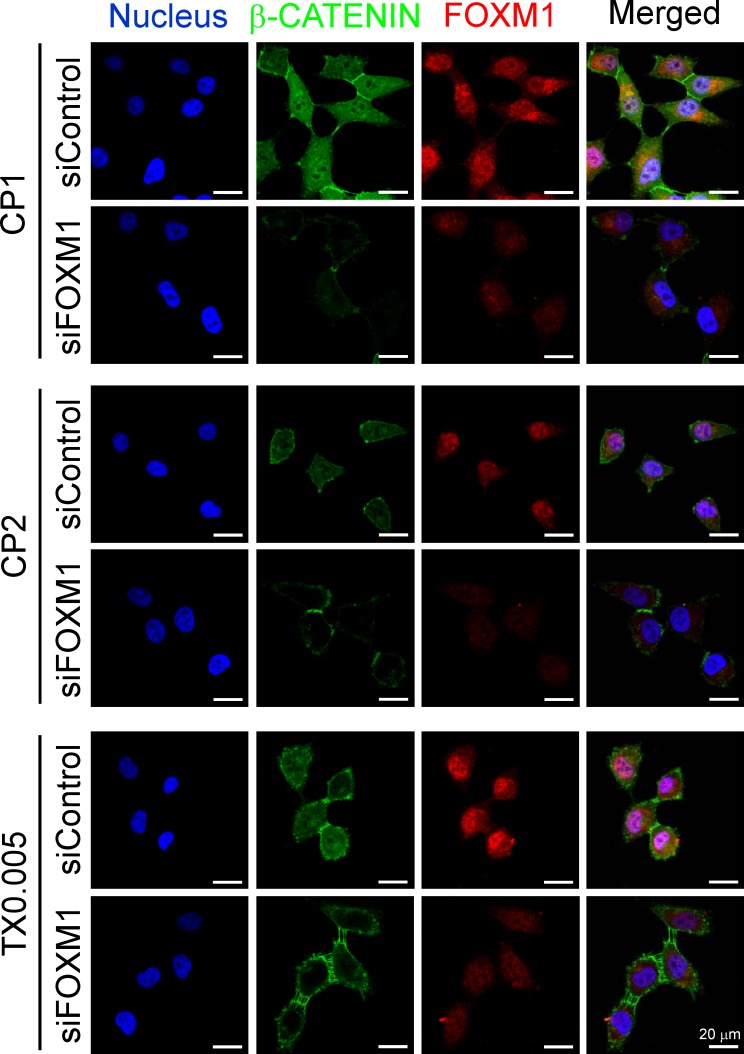
FOXM1 is essential for nuclear localization of β-CATENIN CP1, CP2, and TX0.005 cells received control siRNA (siControl) or FOXM1 siRNA (siFOXM1) for 48 hours. Immunofluorescence staining of the nucleus, β-CATENIN, and FOXM1 in the three cell lines is shown. Representative fluorescence images were taken on a confocal microscope under excitation at 405 nm, 488 nm or 543 nm. Scale bars, 20 μm.

Total β-CATENIN expression and activation (indicated by β-CATENIN dephosphorylation) were higher in both cisplatin-resistant and paclitaxel-resistant IGROV1 cells than parental cells, as was expression of c-MYC, which is encoded by a β-CATENIN target gene (Fig. 7A) Chemoresistant cells had higher nuclear levels of FOXM1 and β-CATENIN than parental cells. Immunofluorescence staining revealed that β-CATENIN was located in the plasma membrane between cell-cell junctions (where it mediates adherence) in parental cells. In chemoresistant cells, it was mostly cytoplasmic or nuclear (Fig. 7B), and nuclear β-CATENIN co-localized with FOXM1 (Fig. 7C). Knockdown of FOXM1 impaired nuclear localization and accumulation of β-CATENIN in both cisplatin-resistant and paclitaxel-resistant IGROV1 cells (Fig. 8).

### WNT/β-CATENIN signaling upregulates the expression of FOXM1

**Figure 9:**
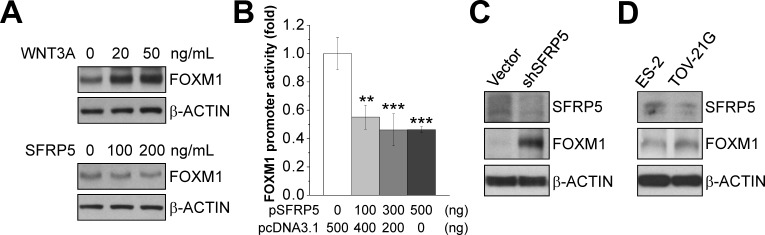
Effect of the WNT/β-CATENIN signaling on FOXM1 expression (A) Western blots of FOXM1 and the internal control β-ACTIN in whole cell lysates of A2780CP70 cells treated for 2 days with different concentrations of WNT3A and SFRP5 peptides. (B) Ad293 cells were transfected with the indicated concentrations of pcDNA3.1 (control vector) or pcDNA3.1 encoding SFRP5 and 500 ng pLuc-FOXM1 (FOXM1 promoter luciferase vector). The cells were harvested two days after transfection, and dual luciferase reporter assays were performed. FOXM1 promoter activity was expressed as the fold change relative to the control (pcDNA3.1). Each bars represents mean ± standard error of the mean from three independent experiments. **: *P* < 0.01; ***: *P* < 0.001. (C and D) Western blots of SFRP5, FOXM1, and the internal control β-ACTIN in Ad293 cells overexpressing a control vector (Vector) or shSFRP5 vector (shSFRP5) (C), and ES-2 and TOV-21G cells (D).

The frizzled-related proteins (SFRPs) are secreted signaling molecules that antagonize WNT/β-CATENIN signaling. We examined the effects of SFRP5 and an activator of WNT/β-CATENIN signaling (WNT3A) on FOXM1 expression in A2780CP70 cells. When added to the culture medium, WNT3A increased FOXM1 expression, and SFRP5 decreased FOXM1 expression, in a dose-dependent manner (Fig. 9A). Overexpression of SFRP5 inhibited the activity of the FOXM1 promoter in a dose-dependent manner, whereas SFRP5 silencing increased FOXM1 expression (Fig. 9B, C). In addition, SFRP5 expression inversely correlated with FOXM1 expression in ES-2 and TOV-21G ovarian cancer cells (Fig. 9D).

### A FOXM1 inhibitor increases sensitivity to cisplatin

**Figure 10:**
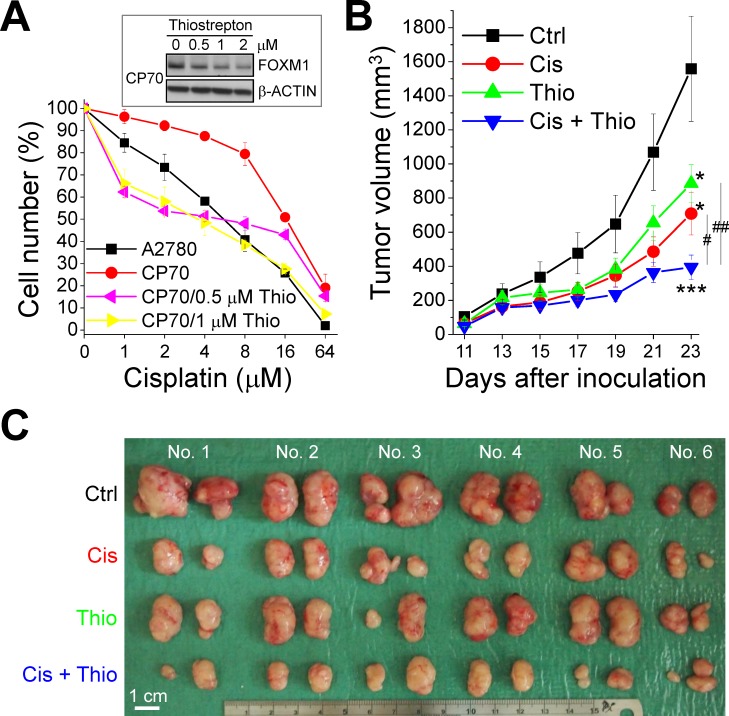
Thiostrepton inhibits the growth of cisplatin-resistant ovarian cancer cells *in vitro* and *in vivo* (A) A2780CP70 (CP70) cells were treated with 0, 0.5, 1, or 2 μM thiostrepton (Thio) and various concentration of cisplatin (Cis) for 48 hours. Cisplatin resistance was determined via MTT assay. The insets show the western blots for FOXM1 and the internal control β-ACTIN. (B and C). CP70 cells were injected subcutaneously into the right scapular region of NOD/SCID mice; mice then received Cis and/or Thio as described in *Materials and Methods*. Tumor growth curves (B) and photographs of isolated tumors (C) are shown. *: significant difference between treated and control (Ctrl) cells (A) or B (mice). *: *P* < 0.05; ***: *P* < 0.001. (B) #: significant difference between single and combined (Cis + Thio) treatments. #: *P* < 0.05; ##: *P* < 0.01.

Our findings suggest that FOXM1 promotes the formation of CSCs and chemoresistance. We asked whether inhibiting FOXM1 activity could be an effective therapeutic strategy in chemoresistant ovarian cancer. As shown in Figs. 10A and S2, the FOXM1 inhibitor thiostrepton decreased the expression of FOXM1 and stem cell markers in a dose-dependent manner in A2780CP70 cells. Pretreatment of cells with thiostrepton sensitized cisplatin-resistant A2780CP70 cells to cisplatin (Fig. 10A; *P* < 0.001, one-way ANOVA). Thiostrepton and cisplatin inhibited the growth of A2780CP70 cells in mouse xenografts compared with the vehicle control, and the combination of cisplatin and thiostrepton was more effective than either alone (Fig. 10B, C; *P* = 0.004, Kruskal-Wallis test).

### Association of FOXM1 with the clinical characteristics

Among 141 EOC samples in tissue microarrays (TMAs) (Table 1), FOXM1 expression determined via immunohistochemistry (IHC) was higher in the serous and endometrioid subtypes and lower in the mucinous subtype and normal ovarian tissue. The relationship between FOXM1 expression in TMA and clinical demographics was examined (Table 2). Higher FOXM1 expression was significantly associated with serous histology (*P* = 0.011; Table 2). The association between FOXM1 expression and clinical outcome was analyzed in 106 newly diagnosed EOC patients at our hospital (Table 3). The patients ranged in age from 27 to 80 years (median, 53 years). Thirty-five (33.0%) patients had FIGO stage I disease, 10 (9.4%) had stage II disease, 55 (51.9%) had stage III disease, and 6 (5.7%) had stage IV disease. Serous, endometrioid, clear cell, and mucinous type histology were found in 52 (49.1%), 15 (14.2%), 28 (26.4%), and 11 (10.4%) patients, respectively. There were 10 (9.4%) well differentiated, 26 (24.5%) moderately differentiated, and 70 (66.0%) poorly differentiated tumors. Optimal cytoreductive surgery was performed in 87 (82.1%) patients. First-line platinum-based regimens included paclitaxel plus carboplatin (n = 88, 83.0%) or cisplatin plus cyclophosphamide (n = 18, 17.0%). Furthermore, 69 patients (65.1%) had platinum-sensitive disease, whereas 37 (34.9%) had platinum-resistant disease. The median follow-up time for all participants was 45.0 months (range, 1 to 218 months). During follow-up, 57 patients (53.8%) developed progressive disease and 44 patients (41.5%) died.

**Table 1:** FOXM1 expressions in epithelial ovarian carcinoma (n=141) and normal ovarian tissue (n=20)

Histology	N	Grade of FOXM1 expression			
		0	1	2	3
Serous	113	2 (1.8)	37 (32.7)	56 (49.6)	18 (15.9)
Mucinous	14	3 (21.4)	8 (57.1)	2 (14.3)	1 (7.1)
Endometrioid	4	-	1 (25.0)	2 (50.0)	1 (25.0)
Clear cell	8	-	5 (62.5)	1 (12.5)	2 (25.0)
Transitional cell	2	-	-	2 (100)	-
Normal ovarian tissue	20	-	20 (100)	-	-

Tissue microarray slides of epithelial ovarian carcinoma and normal ovarian tissue (TMA-OV2085 and TMA-OV809) were obtained from US Biomax, Inc. (Rockville, MD, USA).

**Table 2:** Analysis of FOXM1 expressions in epithelial ovarian carcinoma (n=141)

				FOXM1 expression		
Variable			N	Low*	High	*P*
Age (year)		< 50	74	34 (45.9)	40 (54.1)	0.112
		≥ 50	67	22 (32.8)	45 (67.2)	
Stage		Early	76	28 (36.8)	48 (63.2)	0.451
		Advanced	65	28 (43.1)	37 (56.9)	
Histology		Serous	113	39 (34.5)	74 (65.5)	0.011
		Non-serous	28	17 (60.7)	11 (39.3)	
Tumor grade		1	26	13 (50.0)	13 (50.0)	0.253
		2 & 3	106	40 (37.7)	66 (62.3)	

* Low: FOXM1 staining ≤ grade 1 ; high: FOXM1 staining > grade 1.

Data was analyzed by X^2^ test or *Fisher's exact* test.

Variables available for analysis included age, histopathologic diagnosis (based on World Health Organization criteria), nuclear grade (based on Gynecologic Oncology Group criteria) and disease stage (according to the International Federation of Gynecology and Obstetrics). Tumor grades are not available in 9 cases in TMA-OV809 and TMA-OV2085.

**Table 3:** The correlation between FOXM1 expressions and patient demographics in epithelial ovarian cancer (n=106)

			FOXM1		
	Variable	n	Negative*	Positive	*P*
**All patients (n=106)**					
Age (year)	≤ 53	57	32 (56.1)	25 (43.9)	0.915
	> 53	49	27 (55.1)	22 (44.9 )	
Stage	Early	45	27 (60.0)	18 (40.0)	0.440
	Advanced	61	32 (52.5)	29 (47.5)	
Histology	Serous	52	24 (46.2)	28 (53.8)	0.053
	Non-serous	54	35 (64.8)	19 (35.2)	
Grade	1 & 2	36	23 (63.9)	13 (36.1)	0.221
	3	70	36 (51.4)	34 (48.6)	
Residual tumor	< 1 cm	87	48 (55.2)	39 (44.8)	0.829
	≥ 1 cm	19	11 (57.9)	8 (42.1)	
Chemotherapy regimen	Platinum & paclitaxel	88	47 (53.4)	41 (46.6)	0.302
	Other platinum-based	18	12 (66.7)	6 (33.3)	
PFI	< 12 months	49	22 (44.9)	27 (55.1)	0.060
	≥ 12 months	57	36 (63.2)	21 (36.8)	
Progression	Yes	57	26 (45.6)	31 (54.4)	0.025
	No	49	33 (67.3)	16 (32.7)	
Tumor progression site	Pelvis	11	5 (45.5)	6 (54.5)	0.991
	Peritoneum, extrapelvic	23	9 (39.1)	14 (60.9)	
	Distant metastasis	23	12 (52.2)	11 (47.8)	
**Age ≤ 53 years (n=57)**					
PFI	< 12 months	28	10 (35.7)	18 (64.3)	0.005
	≥ 12 months	29	21 (72.4)	8 (27.6)	
Progression	Yes	32	12 (37.5)	20 (62.5)	0.004
	No	25	19 (76.0)	6 (24.0)	
**Serous histology (n=52)**					
PFI	< 12 months	26	8 (30.8)	18 (69.2)	0.026
	≥ 12 months	26	16 (61.5)	10 (38.5)	
Progression	Yes	33	11 (33.3)	22 (66.7)	0.015
	No	19	13 (68.4)	6 (31.6)	
**Platinum plus paclitaxel chemotherapy (n=88)**					
PFI	< 12 months	39	16 (41.0)	23 (59.0)	0.038
	≥ 12 months	49	31 (63.3)	18 (36.7)	
Progression	Yes	47	19 (40.4)	28 (59.6)	0.009
	No	41	28 (68.3)	13 (31.7)	

* Negative: no FOXM1 staining; Positive: FOXM1 staining ≥ grade 1.

Abbreviation: PFI, progression-free interval

Data was analyzed by X^2^ test or *Fisher's exact* test.

### FOXM1 expression correlates with tumor progression and patient survival

**Figure 11:**
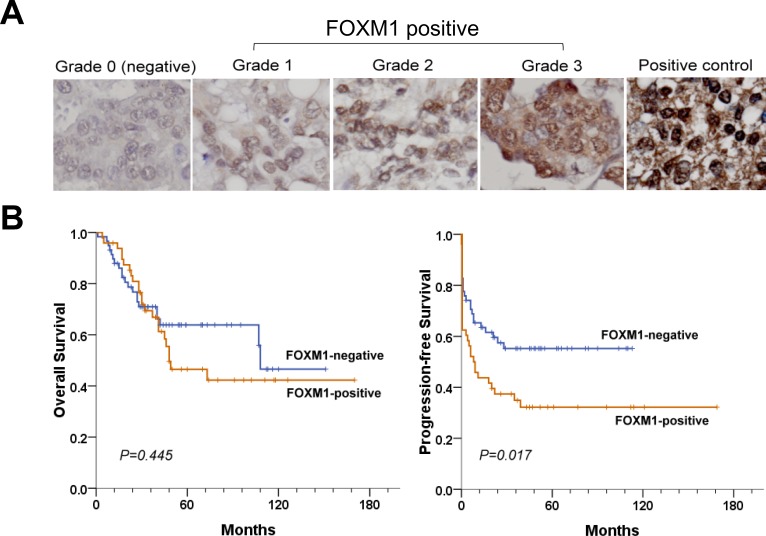
FOXM1 expression correlates with tumor progression and patient survival (A) Immunohistochemical staining of FOXM1 in primary ovarian carcinomas. Pancreatic cancer cells served as the positive control. (B) Kaplan-Meier curves. Survival rates were compared using the log rank test.

The association between FOXM1 expression and treatment outcome was examined (Table 3). Notably, FOXM1 overexpression at diagnosis was significantly associated with tumor progression (*P* = 0.025). FOXM1 was expressed in 27 (55.1%) patients with progression-free intervals (PFIs) < 12 months and 21 patients (36.8%) with PFIs ≥ 12 months (*P* = 0.060). FOXM1-positive tumors were significantly associated with PFIs <12 months in patients less than ≤ 53 years of age, patients with serous histology, and patients receiving platinum-paclitaxel combination chemotherapy (*P* = 0.005, 0.026, and 0.038, respectively). FOXM1-positive tumors were also significantly associated with cancer progression in these three subgroups (*P* = 0.004, 0.015, and 0.009, respectively). The median times to progression and death in the FOXM1-positive group were 9.0 and 45.0 months, respectively. The hazard ratios were 1.72 (95% confidence interval [CI], 1.02 to 2.90; *P* = 0.04) for risk of progression and 1.28 (95% CI, 0.71 to 2.32; *P* = 0.41) for risk of death when compared with the FOXM1-negative group. As shown in Fig. 11, FOXM1-positive patients had significantly shorter progression-free survival (PFS) times than FOXM1-negative patients (*P* = 0.017). However, overall survival (OS) times were not different (*P* = 0.445).

## DISCUSSION

FOXM1 is a proto-oncoprotein that is overexpressed in various types of cancer. Because it modulates tumor growth, angiogenesis, metastasis, apoptosis, DNA damage repair, and tissue regeneration in different types of cancer cells, it may play diverse roles in tumorigenesis. FOXM1 is frequently upregulated in ovarian cancers, most notably (and significantly) in high-grade tumors with aggressive behavior, such as metastasized lymph nodes. Ectopic expression of FOXM1 markedly enhances cell proliferation, migration, and invasion in ovarian cancer cells, and it is antagonized by p53 [31]. Recent analysis of 489 high-grade serous ovarian cancers by the Cancer Genome Atlas consortium indicates that FOXM1 overexpression may be a key, early event driving the growth of epithelial ovarian cancers, especially those with serous pathophysiology. Pathway analyses suggest that NOTCH and FOXM1 signaling contribute to serous ovarian cancer pathophysiology [32]. Moreover, FOXM1 hyperactivity is a consistent feature of epithelial ovarian cancer and contributes to ovarian cancer metastasis as well as proliferation [33].

We identified FOXM1 as a candidate biomarker of chemoresistance and poor outcome in ovarian cancer. The principal findings of our study are that FOXM1 reduces the sensitivity of cells to anti-cancer drugs such as cisplatin and paclitaxel *in vitro* and that FOXM1 overexpression in tumors predicts cancer progression and unfavorable prognosis, especially in young EOC patients, patients with serous histology, and patients receiving platinum-paclitaxel combination chemotherapy. Our results also suggest that chemoresistance induces the EMT and stem cell phenotypes in ovarian cancer cells and thereby enhances invasion and sphere formation ability. Chemoresistance may be induced after exposure to cisplatin and paclitaxel via reciprocal interaction between FOXM1 and the WNT/β-CATENIN signaling pathway, and FOXM1 is a major inducer of the expression, nuclear translocation, and activation of β-CATENIN, as well as a possible inhibitor of hCTR1-mediated cellular import of cisplatin.

Chemotherapy is an important therapeutic strategy for most patients with cancer. An understanding of the molecular mechanisms underlying chemoresistance in patients with aggressive cancers, such as EOC, might aid the design of new treatment strategies. In our study, ovarian cancer cells resistant to cisplatin or paclitaxel showed similar molecular changes, including the upregulation of mesenchymal markers, EMT-associated transcription factors, and stem cell markers and the downregulation of epithelial markers. Moreover, our unpublished findings suggest the possibility of cross-resistance between cisplatin and paclitaxel in a cell-based model. Cisplatin-resistant IGROV1 cells exhibited more pronounced changes in cell morphology and invasive ability than paclitaxel-resistant IGROV1 cells, perhaps because paclitaxel-mediated microtubule polymerization limits the acquisition of the EMT phenotype and invasiveness. Altogether, our findings provide an explanation for the high recurrence rate following first-line cisplatin and paclitaxel chemotherapy, and this should be noted by clinicians.

The cytotoxicity of chemotherapeutic agents in cancer cells is dependent on their uptake and extrusion. Copious clinical data associate the multi-drug resistance (MDR) phenotype in tumors with the overexpression of certain ABC transporters termed MDR proteins [34]. FOXM1 and its downstream DNA damage repair targets RAD51 [35], NBS1 [36], BRIP1 [37], BRCA2, and XRCC1 have been shown to render cells resistant to cisplatin [18], epirubicin [37], herceptin, and paclitaxel [19]. How FOXM1 regulates the transport of chemotherapeutic agents into and out of cancer cells via plasma membrane transporters is, however, unclear. hCTR1 is a transmembrane protein that transports copper and cisplatin into mammalian cells. Our data suggest that FOXM1 may confer cisplatin resistance, at least in part, by inhibiting hCTR1 expression (Fig. 6). This is the first study to demonstrate a direct link between FOXM1 and cisplatin uptake.

The EMT phenotype and CSCs play critical roles in chemoresistance [38, 39]. It has been suggested that the WNT/β-CATENIN and transforming growth factor (TGF-β)/SMAD signaling pathways, acting downstream of FOXM1 targets, drive cancer progression by inducing the EMT. WNT/β-CATENIN target genes can be divided into a stemness/proliferation group and an EMT/dissemination group. β-CATENIN has been shown to activate LEF-1 transcription in regulating the expression of several EMT-related genes during the EMT [40], and FOXM1 has been shown to maintain the nuclear SMAD3/SMAD4 complex [41].

Both FOXM1 and β-CATENIN are potent transcription factors that regulate many aspects of tumorigenesis. The reciprocity of FOXM1 and β-CATENIN signaling may amplify both pathways, which have been shown to exert multiple functions related to drug resistance, EMT, stemness, and migration/invasion. Ovarian cancer cells that gain EMT-related functions and stemness during the development of drug resistance can contribute to cancer recurrence. In this study, we found that upregulation of FOXM1 promoted β-CATENIN nuclear translocation and activation. Conversely, FOXM1 was a downstream target of β-CATENIN. FOXM1 may be a useful target for overcoming the chemoresistance of ovarian cancer cells because it is upstream of multiple cancer-related signaling pathways and amplifies β-CATENIN signaling.

CSCs have emerged as new targets for anti-cancer therapy in addition to non-CSC tumor cells. Elimination of CSCs, which play a major role in drug resistance and disease recurrence, is critical to improving cancer treatment outcomes. Both FOXM1 and β-CATENIN, whose expression was FOXM1-dependent, are CSC markers. Our study demonstrates that the combination of thiostrepton, a FOXM1 inhibitor, and cisplatin significantly decreases the expression of stem cell markers and suppresses tumor formation *in vivo*. It also suggests that inclusion of FOXM1 inhibitors in chemotherapy regimens can help eradicate ovarian CSCs, as well as non-CSC ovarian tumor cells, by overcoming drug resistance.

The development of platinum resistance is a complex molecular process. Predictors of platinum resistance would help identify subgroups at high risk during early cancer progression, and patients should be individually assessed to determine the optimal therapy in terms of tumor recurrence and platinum sensitivity. Our observations that FOXM1 is highly expressed in platinum-resistant cell lines and that FOXM1 overexpression is associated with the progression of serous type EOCs are consistent. They also agree with previous findings linking FOXM1 signaling to serous ovarian cancer pathophysiology [32]. High FOXM1 expression at diagnosis may predict unfavorable PFIs and PFS. Individualized therapies, such as dose-dense first-line chemotherapy [42], paclitaxel maintenance chemotherapy [43], and combination chemotherapy with an anti-angiogenic agent as first-line or maintenance therapy [44], could be implemented to prolong PFIs and improve long-term prognosis. Future clinical trials should be designed to evaluate the usefulness of FOXM1 inhibitors in platinum-based chemotherapy.

## MATERIALS AND METHODS

### Cell lines and reagents

Cells were maintained as adherent monolayers in Dulbecco's modified Eagle's medium (DMEM, Ad293 cells), McCoy's 5A medium (ES-2 cells), 1:1 MCDB105/M199 medium (TOV-21G cells), RPMI 1640 medium (IGROV1, A2780, and A2780CP70 cells), or DMEM/F12 medium (SKOV-3 and BG-1 cells) supplemented with 10% fetal bovine serum under 5% CO_2_ at 37°C. Recombinant human SFRP5 and WNT3a peptides were purchased from R&D Systems.

### Selection of drug-resistant ovarian cancer sublines

Chemoresistant sublines were obtained by exposing ovarian cancer cells to stepwise increases in cisplatin (Sigma-Aldrich) or paclitaxel (Sigma-Aldrich) concentrations. The cells received cisplatin or paclitaxel at the initial concentration for 3 days during a 3-6 week period, allowing for growth recovery between cycles. After the completion of three cycles at the initial concentration, the dose of cisplatin or paclitaxel was doubled and the procedure repeated until the noted drug levels with significant cell death were achieved. The IC_50_ values of the chemoresistant sublines were determined via MTT assays.

### Plasmids, shRNA, siRNA, and transfection

The full-length cDNAs for *FOXM1* and *SFRP5* and the shRNA sequences for FOXM1 and SFRP5 were described previously [45-47]. The sequence of the FOXM1 siRNA (siFOXM1) was 5′-CCUUUCCCUGCACGACAUGtt-3′ (Qiagen). Plasmids, shRNAs, and siRNAs were transfected into ovarian cancer cells using Lipofectamine^TM^ 2000 (Invitrogen), and stable clones were selected with G418 or hygromycin.

### MTT assays

Ovarian cancer cells were seeded at a density of 3 × 10^3^ cells per well in 96-well plates. The following day, the cells received various doses of cisplatin, paclitaxel, or thiostrepton. After treatment, MTT (20 μL, 5 mg/mL; Sigma-Aldrich) was added to each well, and the plates were stored at 37°C for 4 hours. Dimethylsulfoxide (100 μL) was added to each well to lyse the cells. Absorbance was measured at 570 nm.

### Immunofluorescence staining, confocal microscopy, and image analysis

Ovarian cancer cells were grown in glass-bottom dishes overnight, fixed in ice-cold 4% paraformaldehyde for 20 minutes, permeabilized with 0.5% Triton X-100 for 10 minutes, and blocked with SuperBlock® Blocking Buffer (Thermo) for 1 hour at room temperature. Cells were then incubated overnight at 4°C with anti-FOXM1 (Santa Cruz), anti-β-CATENIN, anti-E-CADHERIN, or anti-VIMENTIN (BD) primary antibody and subsequently with Alexa 488 or 543-conjugated secondary antibody (Invitrogen) and the DNA dye Hoechst 33258 (10 mg/mL) for 1 hour at room temperature. Immunofluorescence images were taken on a laser scanning confocal microscope (Olympus FLUOVIEW FV1000) using a 405 nm, 488 nm, or 543 nm laser.

### Western blots

Cell lysates were harvested in ice-cold modified radioimmune precipitation assay buffer containing a protease inhibitor cocktail^TM^ (Roche). Lysates normalized for amount protein were separated on 10% SDS-polyacrylamide gels and electrophoretically transferred to nitrocellulose. The blots were incubated with primary antibodies to E-CADHERIN, VIMENTIN, FIBRONECTIN, β-CATENIN (BD), c-MYC, FOXM1, TFIIB, MYD88, γ-CATENIN, SNAIL1, SLUG, TWIST, hCTR1 (Santa Cruz), BMI1, CD44, NANOG, ALDH-1, OCT-4, NOTCH-1, SFRP5 (Abcam), ZO-1 (Invitrogen), phospho-β-CATENIN, N-CADHERIN (Cell Signaling), or SOX-2 (Sigma). Immunocomplexes were detected with horseradish peroxidase-conjugated IgG and visualized via enhanced chemiluminescence (ECL detection kit, Amersham).

### Dual luciferase assay

The *FOXM1* luciferase reporter construct (pLuc-*FOXM1*) was described previously [8]. Cells were co-transfected with pLuc-*FOXM1* or other gene-specific expression plasmids, and, for normalization of transfection efficiency, p*β-ACTIN*-RL, which contains the Renilla luciferase gene under the control of the human *β-ACTIN* promoter, was used. Luciferase activity was quantified using a dual-luciferase reporter assay system (Promega Corporation).

### Sphere formation assay

Cells were cultured in ultra-low attached plates in serum-free medium containing 5 μg/mL insulin, 0.4% bovine serum albumin, 10 ng/mL basic fibroblast growth factor, and 20 ng/mL human recombinant epidermal growth factor for seven days. The spheres in the suspension cultures were counted on a widefield light microscope.

### *In vitro* transwell invasion assays

The transwell chambers used for invasion assays contained polycarbonate filters (8-μm pore size; BD Biosciences) whose upper surfaces were coated with a growth factor-reduced Matrigel matrix. Medium containing 10% fetal bovine serum was placed in the lower chambers to act as a chemoattractant. Cells (2 × 10^4^ in 500 μL serum-free medium) were placed in the upper chamber and incubated at 37°C for 20 hours. The cells that penetrated the Matrigel-coated filter were counted in 15 randomly selected fields, and the mean number of cells per field was recorded. Each assay was performed on duplicate filters, and each experiment was repeated twice.

### Subcutaneous tumor growth in xenografts

Ovarian cancer cells (1 × 10^6^ in 0.1 mL Hank's balanced salt solution) were injected subcutaneously into the right scapular region of pathogen-free female NOD/SCID mice. The length (L) and width (W) of the resulting tumors were measured using calipers, and tumor size was calculated as W^2^ × L/2.

### Drug preparation and administration

Cisplatin was diluted in normal saline, and thiostrepton was dissolved in N,N-dimethylacetamide, polyethylene glycol 400, and Tween 80 at a 2:7:1 v/v/v ratio. Cisplatin and thiostrepton were intravenously injected into mice seven days after tumor cell inoculation at 3 mg/kg and 50 mg/kg mouse body weight, respectively. Cisplatin was injected every three days, and thiostrepton was injected daily.

### TMAs

Formaldehyde-fixed, paraffin-embedded TMA slides of primary ovarian carcinoma and normal ovarian tissues [TMA-OV2085 (208 cores/104 cases) and TMA-OV809 (80 cores/80 cases)] were purchased from US Biomax, Inc. EOC patients and 20 subjects with normal ovarian epithelia were included in the early stage of our analyses. FOXM1 expression determined via IHC was compared in the various groups based on the provided patient characteristics.

### EOC patients and tissue samples

Consecutive patients diagnosed with EOC between April 1993 and October 2010 at National Cheng Kung University Hospital were included in our study. These patients underwent comprehensive staging or cytoreductive surgery with adjuvant chemotherapy consisting of platinum-based chemotherapeutic agents. Patients who received primary surgery elsewhere or who did not receive adjuvant chemotherapy or did not provide written informed consent were excluded. Cancer progression was defined according to the objective Response Evaluation Criteria in Solid Tumors (RECIST) version 1.1 or the Gynecologic Cancer Intergroup definition for CA125 progression. EOC patients were clinically defined as “resistant,” “partially sensitive,” and “sensitive” to platinum on the basis of their PFIs (< 6, 6–12, and > 12 months, respectively) [48, 49]. Both PFS and OS were calculated from the date of diagnosis. OS was measured to the date of death from any cause; data on survivors were censored on the date they were last known to be alive. PFS was measured to the date of first clinical progression or death from any cause, unless the patient was progression-free at the time of last contact, in which case PFI was measured to the date of last contact. All participants were followed up after treatment, and the date of the latest record retrieved was February 28, 2014. Medical records and pathological slides were reviewed to provide information on patient demographics, clinical characteristics, pathological diagnoses, and outcomes. The research protocol was approved by our institutional review board. Cancerous tissues from ovarian sites were fixed in formaldehyde, embedded in paraffin, and sectioned (4 μm thickness). Sections were examined for confirmation of histopathology following routine staining with hematoxylin and eosin.

### IHC and grading of FOXM1 expression levels

FOXM1 protein expression was assessed via IHC as previously described [27]. Briefly, IHC was performed on formaldehyde-fixed, deparaffinized tissue sections after microwave-enhanced epitope retrieval according to the standard automated IHC procedure (Ventana XT autostainer). Sections were incubated with mouse monoclonal anti-FOXM1 antibody (Abnova Corporation) was applied at a dilution of 1:50 phosphate-buffered saline (negative control). Staining was scored by an investigator unaware of the corresponding clinical outcome as follows: grade 0 = negative; grade 1 = weak; grade 2 = moderate; and grade 3 = strong (Fig. 11A). Grades 1-3 were considered “positive” for expression, while grade 0 was considered “negative”.

### Statistics

Data were analyzed using the Statistical Package for the Social Sciences software, version 17.0, for Windows (SPSS Inc.). Student's *t*-test was used to compare two groups consisting of normally distributed interval data. One-way ANOVA and Kruskal-Wallis tests were used to compare three or more groups when interval data was normally distributed and not necessarily normally distributed, respectively. Frequency distributions between categorical variables were compared using the chi-square test and Fisher's exact method. OS and PFS were estimated according to the Kaplan-Meier method and compared using the log-rank test. To assess potential associations between FOXM1 overexpression and cancer progression or death, hazard ratio and confidence intervals were estimated by Cox proportional hazard regression models. *P* < 0.05 (two-sided) was considered significant.

## SUPPLEMENTARY MATERIAL, FIGURES


