# Supplementary material for: FOXM1 confers to epithelial-mesenchymal transition, stemness and chemoresistance in epithelial ovarian carcinoma cells
Source: Oncotarget. 2014 Dec 10;6(4):2349–65. doi: 10.18632/oncotarget.2957 (PMC4385856; doi:10.18632/oncotarget.2957)
Supplement: Supplementary file 1 [file oncotarget-06-2349-s001.pdf]

FOXM1 confers to epithelial-mesenchymal transition, stemness and chemoresistance in epithelial ovarian carcinoma cells

Supplementary Material

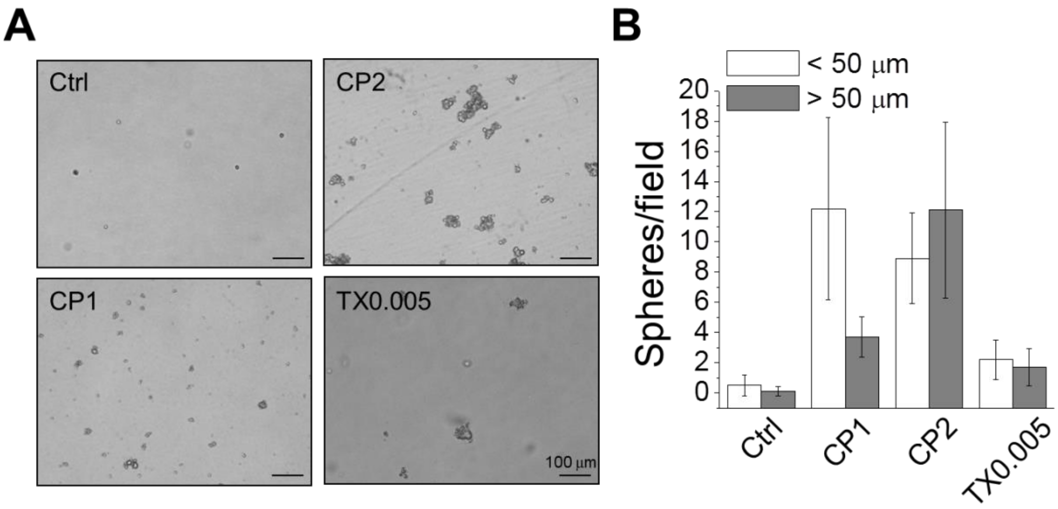

Supplementary Figure S1: Number and size of spheres formed in chemoresistant IGROV1 sublines. The sphere formation assay was performed in parental (Ctrl) and cisplatin-resistant (CP1 and CP2) and paclitaxel-resistant (TX0.005) IGROV1 cells. (A) Representative phase contrast images of spheres were obtained on a widefield microscope. Scale bars, 100 µm. (B) Quantification of sphere formation in each cell line using low-power field magnification. Spheres were classified as large (> 50 µm) and small sizes (< 50 µm). Each bar represents mean ± standard error of the mean from two independent experiments and at least 20 different fields

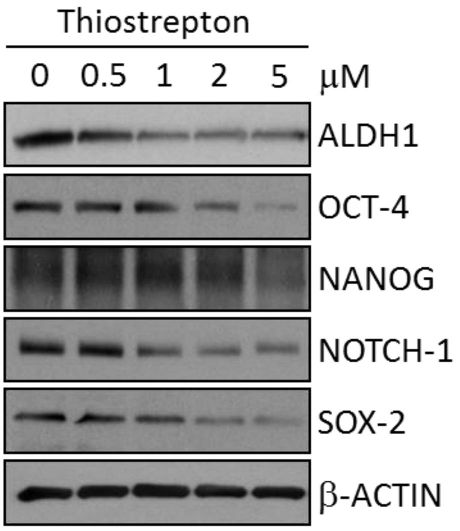

Supplementary Figure S2: Effect of the FOXM1 inhibitor thiostrepton on the expression of stem cell markers. Whole cell lysates of A2780CP70 cells treated with various concentrations of thiostrepton for 48 hours were western blotted with antibodies to the indicated proteins. β-ACTIN is the internal control.
